# Minocycline-induced hypersensitivity syndrome presenting with meningitis and brain edema: a case report

**DOI:** 10.1186/1752-1947-1-22

**Published:** 2007-05-18

**Authors:** Nicolas Lefebvre, Emmanuel Forestier, David Farhi, Mohseni Zadeh Mahsa, Véronique Remy, Olivier Lesens, Daniel Christmann, Yves Hansmann

**Affiliations:** 1 Department of Infectious Diseases and Tropical medicine, Teaching Hospital, Strasbourg, France; 2 Department of Dermatology, Tarnier Hospital, Paris, France

## Abstract

**Background:**

Hypersentivity Syndrome (HS) may be a life-threatening condition. It frequently presents with fever, rash, eosinophilia and systemic manifestations. Mortality can be as high as 10% and is primarily due to hepatic failure. We describe what we believe to be the first case of minocycline-induced HS with accompanying lymphocytic meningitis and cerebral edema reported in the literature.

**Case presentation:**

A 31-year-old HIV-positive female of African origin presented with acute fever, lymphocytic meningitis, brain edema, rash, eosinophilia, and cytolytic hepatitis. She had been started on minocycline for inflammatory acne 21 days prior to the onset of symptoms. HS was diagnosed clinically and after exclusion of infectious causes. Minocycline was withdrawn and steroids were administered from the second day after presentation because of the severity of the symptoms. All signs resolved by the seventh day and steroids were tailed off over a period of 8 months.

**Conclusion:**

Clinicians should maintain a high index of suspicion for serious adverse reactions to minocycline including lymphocytic meningitis and cerebral edema among HIV-positive patients, especially if they are of African origin. Safer alternatives should be considered for treatment of acne vulgaris. Early recognition of the symptoms and prompt withdrawal of the drug are important to improve the outcome.

## Background

Hypersentivity Syndrome (HS) is a rare and life-threatening form of drug reaction [1]. Usual presentation includes fever, rash, eosinophilia and systemic manifestations. Mortality may be as high as 10% and is primarily due to hepatic failure [2]. HS is frequently associated with the use of sulfonamides, allopurinol, terbinafine, minocycline, and antiretroviral therapy [3].

Minocycline is widely prescribed for the treatment of inflammatory forms of *acne vulgaris *[4]. Although it is considered to be a safe drug [2], it has been reported to cause serious adverse events such as hepatitis, auto-immune syndrome and HS [1,5]. To our knowledge, lymphocytic meningitis and brain edema associated with minocycline-induced HS have not been reported in the literature. This presentation, which is probably under-recognized, may lead to a diagnostic delay. We report herein one case.

## Case presentation

A 31-year-old female native of Africa was hospitalized with fever, weakness, nausea, headache, facial edema, and rash, for 4 days. She had been diagnosed as HIV positive 2 years previously. CD4 cell count was between 300 and 400 cells/mm^3^, viral load was near 150,000 copies/mm^3^, both steady for several weeks. She had no other relevant medical history, and was on no treatment for HIV. Three weeks before the onset of the symptoms, she had been started on oral minocycline to control an inflammatory form of *acne vulgaris*.

At admittance she was unwell and vital signs were: blood pressure 100/60, temperature 40°C, heart rate 120 beats/min., respiratory rate 22/min., and oxygen saturation 97% (room air). On physical examination, she had multiple erythematous, oedematous and coalescing plaques on the upper trunk, and on proximal segments of the limbs. Her palms and soles were erythematous. She also had injection of the sclera, and edema of the eyelids. Buccal and genital mucous membranes were unaffected. Palpation revealed enlarged tender lymph nodes at all sites, but no accompanying hepatomegaly or splenomegaly. Cardiovascular and pulmonary systems were normal, with no sign of septic shock. Neurological examination was normal.

Laboratory tests revealed the following values: leucocytosis (24×10^9^/L), eosinophilia (2.8×10^9^/L), neutrophilia (8×10^9^/L) without lymphocytosis (2.8×10^9^/L), elevated C-Reactive Protein (17 mg/L) and cytolytic hepatitis (ALT 160 U/L, AST 106 U/L, lactate deshydrogenase 1400 U/L (LDH)). Multiple blood culture and bacteriological analysis of urine were sterile. Serological investigation for viral and bacterial agents, repeated over two weeks showed no sign of recent infection (*Borrelia burgdorferi*, *Mycoplasma sp*., *Chlamydiae sp., Legionella sp*., Epstein-Barr virus, cytomegalovirus, hepatitis A, B and C viruses, measles, rubella, parvovirus B19, coxsackievirus, echovirus, VZV, HTLV1, HSV, toxoplasmosis). The antinuclear antibodies were negative. Chest radiography and echocardiography showed no abnormalities, however cerebral CT-scan showed a diffuse cerebral edema (figure). Electroencephalogram was normal. The lumbar puncture revealed lymphocytic meningitis with 60 cells/microliter (lymphocytes: 90%, protein level: 0.80 g/L, glucose level: 0.56 g/L). No microbial agents were found in the cerebrospinal fluid (cryptococcal antigen and culture, culture for bacteriological agents, and polymerase chain reaction (PCR) for echovirus, coxackies virus, CMV, HSV, VZV). PCR for HIV in cerebrospinal fluid was not performed. Histopathology of a skin biopsy sample showed a lymphocytic infiltrate into the dermo-epidermis junction with edema, resulting in a blister detachment. This aspect was of a lichenoid toxiderma. Lymph node biopsy was not performed.

Minocycline was discontinued on the fourth day after the onset of the symptoms. Two days after minocycline withdrawal, treatment with steroids was introduced (methylprednisolone 60 mg daily) because of the severity of the symptoms. The patient improved quickly after steroid administration. Temperature dropped to 37°C within three days. Within one week the eruption cleared and lymphadenopathies disappeared. Biological abnormalities (eosinophilia, liver enzyme elevation) resolved within three days. A second brain CT-scan, undertaken 14 days after the onset of the symptoms, was normal. Lumbar puncture was not repeated. The steroids were steadily tailed off (5 mg per week) but a relapse occurred on week 6, when on a dose of 30 mg per day. This relapse was under the form of a transient and isolated generalized pruritus with no cutaneous nor neurological signs. Skin tests were not carried out due to the potential risk of severe reaction. The patient was weaned off steroids over a period of 8 months and was free of symptoms at discharged form care.

## Discussion

Minocycline is widely prescribed for *acne vulgaris *[4]. Minor adverse effects including nausea, vomiting, dizziness, photosensitivity and skin eruption have been described [2]. However, acute and severe reactions such as HS, autoimmune disorders, serum sickness like reaction, and pseudotumor cerebri syndrome have also been reported [1,5-8].

The case described had several features suggesting a diagnosis of HS secondary to minocycline treatment. The history was characteristic (interval of 3 weeks between the introduction of the drug and the symptoms) and the condition resolved promptly following cessation of minocycline. Moreover, differential diagnoses due to the most likely infectious diseases were excluded and no evidence of pseudotumor cerebri syndrome, as it may be observed with minocycline, was found. While lymphocytic meningitis and cerebral edema have not been described in association with minocycline-induced HS, they have been reported following use of allopurinol [9].

HS to minocycline is a nosological entity also called DRESS syndrome (Drug Reaction with Eosinophilia and Systemic Symptoms). It occurs within 3 to 4 weeks after starting therapy, usually in a young patient (21.2 ± 1.8 years-old) [1,5]. As in our patient, HIV-infection and black African origin have been suggested as risk factors for minocycline HS [3]. Clinical features include three major elements: (1) high fever with asthenia; (2) acute generalized cutaneous signs often polymorphous (maculo-papular rash sometimes morbiliform, exanthematous, or multiform); facial edema is evocative and intense pruritus is common; and (3) multivisceral involvement [3,5,10]. The most common visceral signs are enlargement of the lymph nodes, hepatomegaly and splenomegaly [1,5]. Severe reactions may lead to hepatitis, pulmonary infiltrates with eosinophilia, myocarditis and interstitial nephritis [1,8]. In 1996, among 13 cases of HS reaction due to minocycline, none had cerebral involvement [1].

Suggestive blood chemistry includes eosinophilia (often over 1.5×10^9^/L), cytolytic hepatitis (from mild elevation of liver enzyme to severe liver failure), LDH elevation and hemolytic anaemia [1,10]. The skin biopsy may show a non specific lymphocytic infiltrate or lichenoid interface dermatitis [1]. The treatment is usually limited to the withdrawal of minocycline, which is usually followed by symptomatic relief [5]. Steroids should be restricted to severe case with threatening renal, liver or lung involvement. They should be used with caution because dependence to steroid is frequent and rebound of the condition may follow their withdrawal, as observed in this case report.

## Conclusion

As minocycline is a widely used drug, clinicians should be aware of the risk of HS, even in the presence of neurological abnormalities. Early warning signs include an acute rash with fever, eosinophilia and elevated liver enzymes. The drug should be immediately and definitively withdrawn for the patient. If used, minocycline should be strictly monitored, especially in African or HIV-infected patients.

## Competing interests

The author(s) declare that they have no competing interests.

## Authors' contributions

NL: participated in patient management, diagnosis and drafted the manuscript.

EF, DF, MMZ: participated in patient management, reviewing the literature and helped to draft the manuscript.

OL, VR, YH, DC: helped in patient management and made final diagnosis.

All authors read and approved the final manuscript.

**Figure 1:**
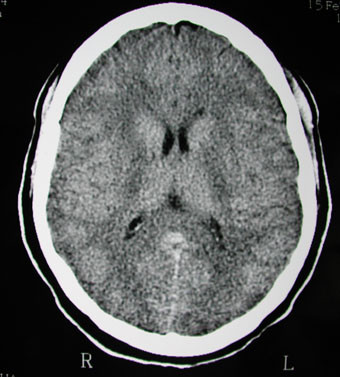
CT scan showing diffuse cerebral edema in a young woman with hypersensitivity syndrome due to minocycline.
